# “I’m still not over feeling so isolated”: Métis women, Two-Spirit, and gender-diverse people’s experiences of the COVID-19 pandemic

**DOI:** 10.17269/s41997-023-00849-3

**Published:** 2024-01-17

**Authors:** Carly Jones, Monique D. Auger, Willow Paul, Renée Monchalin

**Affiliations:** 1 School of Social Work, University of Victoria, Victoria, BC Canada; 2 School of Public Health and Social Policy, University of Victoria, Victoria, BC Canada; 3 Dalla Lana School of Public Health, University of Toronto, Toronto, ON Canada; 4 Well Living House, Unity Health Toronto, Li Ka Shing Knowledge Institute, St. Michael’s Hospital, Toronto, ON Canada

**Keywords:** Métis, Urban, Health access, COVID-19, Health, Indigenous, Cultural safety, Métis, zone urbaine, accès à la santé, COVID-19, santé, autochtones, sécurisation culturelle

## Abstract

**Objectives:**

The aim of this study was to explore and learn from the experiences of Métis women, Two-Spirit, and gender-diverse people accessing health and social services in Victoria, British Columbia, during the COVID-19 pandemic.

**Methods:**

This paper comes from a larger study exploring Métis women, Two-Spirit, and gender-diverse people’s experiences accessing health and social services in Victoria. Using a by-and-for Métis approach that employed a conversational interview method, we conducted interviews with Métis women, Two-Spirit, and gender-diverse people who lived in and/or accessed services in Victoria in December 2020 and January 2021. This paper focuses specifically on data addressing how COVID-19 impacted these participants.

**Results:**

A total of 24 Métis women, Two-Spirit, and gender-diverse people participated in the study. Overall, three themes specific to COVID-19 were identified. First, participants described the detrimental impacts of COVID-19 on their ability to connect with their Métis community and practice their culture, as well as their overall feelings of isolation. Second, participants highlighted some of the ways that COVID-19 has exacerbated existing barriers to culturally safe healthcare. Last, participants spoke about the mixed economic impacts that COVID-19 has had for them, sharing insight into the ways in which gender, in particular, has shaped their financial instability.

**Conclusion:**

Improving access to culturally safe health and social services by incorporating the experiences and expertise of Métis women, Two-Spirit, and gender-diverse people is crucial to mitigating the disproportional negative impacts of the pandemic and improving overall health outcomes within Métis communities across Canada.

**Supplementary information:**

The online version contains supplementary material available at 10.17269/s41997-023-00849-3.

## Résumé

**Objectifs:**

Explorer les expériences de femmes métisses et de personnes métisses bispirituelles et de diverses identités de genre ayant accédé aux services sociaux et de santé à Victoria (Colombie-Britannique) pendant la pandémie de COVID-19, et en tirer des leçons.

**Méthode:**

Cet article vient d’une vaste étude sur les expériences de femmes métisses et de personnes métisses bispirituelles et de diverses identités de genre ayant accédé aux services sociaux et de santé à Victoria. À l’aide d’une démarche par et pour les personnes métisses qui a fait appel à une méthode d’entrevue directe, nous avons mené des entrevues avec des femmes métisses et des personnes bispirituelles et de diverses identités de genre ayant vécu à Victoria en décembre 2020 et janvier 2021 et/ou accédé à des services dans cette ville durant cette période. Le présent article porte spécifiquement sur les données liées aux incidences de la COVID-19 chez ces personnes.

**Résultats:**

En tout, 24 femmes et personnes métisses bispirituelles et de diverses identités de genre ont participé à l’étude. Dans l’ensemble, trois aspects relatifs à la COVID-19 sont ressortis des données. Premièrement, les personnes participantes ont décrit les effets préjudiciables de la COVID-19 sur leur capacité de rester en lien avec leur communauté métisse et de pratiquer leur culture, ainsi que leurs sentiments d’isolement en général. Deuxièmement, elles ont souligné certaines des façons dont la COVID-19 a exacerbé les barrières existantes à l’accès aux soins de santé culturellement sûrs. Enfin, les personnes participantes ont parlé des retombées économiques mitigées de la COVID-19 dans leur cas, et elles ont partagé leurs idées sur le rôle du genre, en particulier, dans leur instabilité financière.

**Conclusion:**

Pour atténuer les effets préjudiciables disproportionnés de la pandémie et améliorer les résultats cliniques globaux au sein des communautés métisses du Canada, il est essentiel d’améliorer l’accès aux services sociaux et de santé culturellement sûrs en y intégrant les expériences et le savoir-faire de femmes métisses et de personnes métisses bispirituelles et de diverses identités de genre.

## Introduction

With distinct historical, cultural, and linguistic traits, the Métis are a recognized group of Aboriginal people within the *Constitution Act*, 1982. While Métis-specific health and wellness remain a relatively overlooked area within research (Gmitroski et al., 2023), studies have shown that Métis people experience a number of inequalities in relation to their determinants of health, as well as concerning disparities in health outcomes when compared to non-Indigenous Canadians (Gmitroski et al., 2023; Loppie & Wien, 2022). Specifically, literature suggests that Métis people face significant barriers to accessing culturally safe health services—with a lack of places where Métis people can access their care while feeling respected, and safe to openly identify as Métis (Auger, 2019; Monchalin et al., 2020; Wesche, 2013). It is well known that such barriers contribute to negative health outcomes for Métis people, as well as for other Indigenous peoples in Canada (Loppie & Wien, 2022; Wesche, 2013).

The ongoing COVID-19 pandemic has imposed new and exacerbated challenges in accessing culturally safe health and social services for Métis people (Addressing Racism Review, 2021; Jones et al., 2020). For example, Métis women in British Columbia (BC) reported experiencing disproportionate effects on their health and well-being during the pandemic, and increased difficulty accessing healthcare compared to non-Indigenous BC residents (Addressing Racism Review, 2021). These disparities are rooted in colonialism, including historical and ongoing assimilative and racist policies imposed onto First Nations, Métis, and Inuit Peoples (Loppie & Wien, 2022; Macdougall, 2017).

With diverse roots in historic Métis communities, Métis people live across Canada, including BC. Recent census data illustrate that there are over 97,000 self-identified Métis people living across the province, including over 7000 who call Victoria, BC, home (Statistics Canada, 2017). Recognizing the need for Métis voices to reflect their experiences about accessing health and social services, we hosted conversational interviews with 24 Métis community members in Greater Victoria, BC. Participants included women, Two-Spirit, and gender-diverse people who self-identify as Métis. While not specific to Métis communities, the term “Two-Spirit” is used within some Indigenous communities, encompassing specific cultural, spiritual, sexual, and gender roles and teachings. This study touched on a number of topics related to Métis culture and identity (Auger et al., 2022); experiences of racism and discrimination in healthcare (Paul et al., 2023); and recommendations for making healthcare more accessible and safe for Métis people (Monchalin et al., 2022). Arising from the same study, this paper specifically shares their stories about accessing health and social services during the COVID-19 pandemic.

## Background

Pandemics such as COVID-19 are not an unprecedented experience for Métis people in Canada. Alongside the numerous injustices collectively experienced by Métis people since the nineteenth century, many Métis families have been impacted by infectious diseases and epidemics (National Aboriginal Health Organization, 2008). In the nineteenth century, First Nations and Métis communities faced prolonged and severe outbreaks of various contagious diseases, including scarlet fever, smallpox, measles, and tuberculosis (Daschuk, 2019; Ward & Macdonald, 2021). Recent public health emergencies, such as the H1N1 influenza outbreak in 2009 and the ongoing opioid crisis, have disproportionately impacted Indigenous communities in Canada, including the Métis (Addressing Racism Review, 2021; Ward & Macdonald, 2021). The compounding impacts of centuries of systemic racism, assimilative policies, and public health emergencies have resulted in immeasurable loss and devastation of not only people, but also land, culture, language, and knowledge (Macdougall, 2017; Ward & Macdonald, 2021). These collective experiences—situated within the overlapping categories of historic and intergenerational trauma—continue to impact the health of Métis people (Driedger et al., 2015). Specifically, Métis women, Two-Spirit, and gender-diverse people often hold overlapping stigmatizing identities, rooted in colonialism, sexism, classism, and racism. These structures continue to shape the health of Métis women, Two-Spirit, and gender-diverse people across Canada (Jones et al., 2020; Monchalin et al., 2020; Ward & Macdonald, 2021).

## Methods

Our larger study from which this paper is drawn takes a by-and-for Métis approach using conversational interviews with self-identified Métis women, Two-Spirit, and gender-diverse people who live in and/or access health and social services in Victoria, BC. The research team consisted of four Métis women—all co-authors of this paper—who worked collaboratively across each stage of the research. The initial idea for the study is rooted in RM’s doctoral research with Métis women in Toronto. After RM moved to Victoria for a faculty position, she met MA—a member of the Métis Nation of Greater Victoria, and a prospective doctoral student. Over tea, they discussed what they perceived to be similarities in terms of access to healthcare for Métis people in two diverse urban settings. After receiving an Insight Development Grant from the Social Sciences and Humanities Research Council, RM and MA spoke with a local and well-respected Métis Elder living in Victoria, who provided valuable guidance on the overall research goals, the interview guide, and the plans for recruiting Métis community members. The funding also allowed for two Métis research assistants—CJ and WP—to join the research team. The interview guide was first drafted from RM’s doctoral research, but was adjusted based on the feedback of local community members, including the Elder and MA. The final interview guide included 10 open-ended questions about Métis identity and experiences accessing health and social services in Victoria. The interview guide included a question asking participants how COVID-19 has impacted them.

### Recruitment and ethics

The study received ethics approval by the Research Ethics Board at the University of Victoria. Participants were recruited via a recruitment poster featuring artwork from a local Métis Two-Spirit artist, Lynette La Fontaine, which was shared through social media. Each of the researchers also reached out to their own kinship networks and online community groups to share the opportunity to participate in the research. The poster included images of Lynette’s traditional Métis beadwork, as well as a description of the research, study criteria, honorarium ($95), and contact information for the research team. Potential participants contacted one of the project’s research assistants (WP), who answered questions about the study and screened each potential participant for eligibility. Participants who identified as Métis women, Two-Spirit, and gender-diverse people, and who lived in or accessed health services in Victoria were invited to participate in the study. We used purposive sampling to recruit people within these gender and cultural categories; we did not tailor our sampling to ensure full representation across all gender identities. Participants were selected on a “first-come, first-considered” basis, meaning that those who expressed interest and were available were invited to participate until we reached our recruitment goal. Interviews were scheduled over Zoom or the telephone, in accordance with the provincial COVID-19 public health protocols. Before commencing the interview, the research assistants completed the informed consent process. A total of 24 Métis women, Two-Spirit, and gender-diverse community members participated in the study.

### Data collection

One-on-one conversational interviews with participants were completed by two Métis Research Assistants during December 2020 and January 2021. An interview guide for this study was developed in consultation with a local Métis Elder. The choice of a conversational interviewing method was significant to our approach because it is congruent with Métis ways of gathering and sharing knowledge through storytelling and informal kitchen-table conversations (Flaminio et al., 2020; Kovach, 2020).

### Data management

Conversations were electronically recorded and transcribed by a third-party transcriptionist. Data were stored on a University of Victoria password-protected and encrypted file, and were de-identified to respect participant confidentiality. Transcripts were mailed to the respective participants to verify their transcripts were written correctly and accurately reflected their experiences. Joining the transcripts was a thank-you card designed by Lynette La Fontaine, along with a COVID-19 face mask designed either by Lynette or by Anishinaabe artist Lesley Hampton. Upon request by the participants, verification, corrections, or requested deletions were integrated into the transcripts.

Throughout this project, our team of Métis women researchers has strived to reflect and honour the stories shared with us in this project, as we understand that the process of sharing stories for Métis people can contribute to healing; as such, Métis scholars have compared stories to medicine (Richardson, 2016). In recognition of the special nature of stories, we selected Métis plant medicines that can be found on Vancouver Island (Table 1) as identifiers, rather than assigning participants a number. Participants were asked if they were happy to use the Michif medicine name in association with their stories, or if they preferred to use a different name. Table 1 includes the list of Michif plant medicines and their English translations. These names can be found alongside the stories shared in the results of this paper.

**Table 1:** Michif medicines used as participant pseudonyms

Michif medicine	English translation
lii shaadroon	Nettle
plaanten	Broadleaf plantain
sasperal	Wild sarsparilla
li sayd	Cedar
enn fleur di pwayzoon	Hemlock
la haarroozh	Red willow
aen nipinet	Spruce
la gratelle	Balsam
lii tii’d mashkek	Labrador tea
li pchi boom	Wild mint
lii bon tiiroozh	Rosehip
enn rooz faroosh	Wild rose
zayon faroosh	Wild onion
lii paabinaan	Cranberry
lii grenn bleu	Blueberry
lii groo zel	Gooseberry
lii frayz	Strawberry
li pisaanlii	Dandelion
bouquets rouge	Fireweed
li tabaa	Tobacco
li grachaw	Burdock
li kors di shenn	Oak bark
enn fleur daan loo	Waterlily

### Analysis and interpretation

A holistic coding method was applied to interview transcripts using NVivo software (QSR International, 2018; Saldaña, 2015). The four authors collaboratively developed a codebook based on initial review of the transcripts. The transcripts were subsequently coded by the project RAs in close collaboration with all authors. Following this, a final thematic analysis (Braun & Clarke, 2006) was conducted by the authors. The authors met weekly to ensure there was group consensus and shared understanding of the identified themes. Following this process, the team developed a community report encompassing all findings from the larger study—featuring the stories shared by the Métis participants. This report was shared with project partners and participants for their feedback. With participant support, the report was sent to Métis communities and local organizations for distribution to service providers and community members. From the larger study, which involved a full analysis of the data, each team member worked with a specific area of findings to develop manuscripts for further, targeted dissemination. Stories related to COVID-19, which were prominently featured across the interviews, are shared in this specific paper.

## Results

This section highlights some of the unique experiences of Métis women, Two-Spirit, and gender-diverse people during the COVID-19 pandemic. The first section shares the ways in which participants were impacted during the pandemic in terms of their connection to culture and community, with a focus on notions of belonging. The second theme addresses barriers to safe healthcare, both from a Métis lens as well as through gender-based analysis. The final section shares the diverse perspectives around economic impacts at a personal and family level for Métis women, Two-Spirit, and gender-diverse people with a look at the unique positionalities of the participants.

### Impacts on belonging, connection, and relationship

Participants often shared that the COVID-19 pandemic has imposed significant barriers to Métis gathering practices and disrupted processes of (re)connecting to their Métis identity, family, community, and culture. Many stories described how the COVID-19 pandemic and the unintended impacts of COVID-19 public health measures have strained their sense of identity and belonging to community in Victoria. When sharing their experiences of being unable to gather in community, practice ceremony, or access the land to gather traditional medicines due to COVID-19, the participants illustrate how connection to community and culture impacts Métis wellness. For example, *lii pchi boom* shared:

It’s been difficult when you want to go out on the land, and that’s a place of restoration to nourish your spirit, and there’s not always the ability to do that or to get medicines... I like to gather with people, I would love to be doing beadwork with folks, I would love to be sitting and doing art or just having dinner and sharing food, and that’s something that we don’t have the ability to do right now. (*lii pchi boom*)

This quote is similar to one from *li pisaanlii*, who goes further to describe how a lack of connection with community has impacted her health since the onset of the pandemic:

COVID-19 has prevented any opportunities for me to engage in a really meaningful way with people that I love and that I care and whose relationship I want to nurture. So I think that’s been really tough on a personal level, not being able to come back to Victoria and to nurture relationships that I want to so that I can feel a sense of belonging and, community here…I don’t sleep amazingly. You know, I’m really tired and I have a hard time focusing at times. It’s hard to tease out whether that’s because of COVID… but ultimately it’s just this lack of belonging and connection with community I think that is at the root for me. (*li pisaanlii*)

Notable from the above excerpt is the participants’ emphasis on relationships. While separation from significant relationships has been a common experience for many people living in Canada throughout the pandemic, Métis participants highlighted that the impacts of COVID-19 on Métis kinship relationships are especially dire, given that many Métis people are already navigating disconnection from family, culture, and community due to ongoing impacts of colonialism. For example, *enn fleur di pwayzoon* articulated how COVID-19 has impacted her health as a Métis survivor of the colonial child welfare system:

One thing it’s really impacted me through being isolated more, which has just been very unhealthy for me. As somebody who grew up in the foster care system and was neglected a lot, and put into isolation a lot, it’s not healthy for me to be isolated. (*enn fleur di pwayzoon*)

The experiences of social isolation during the pandemic described by *enn fleur di pwayzoon* were echoed across many conversations. Social isolation seemed to present a heightened challenge for Métis women, Two-Spirit, and gender-diverse people who lived alone or were new to Victoria. For example, *bouquets rouge* shared:

It was very hard first semester ’cause I was living by myself and I couldn’t make any new friends. None of my family is here, so I felt very isolated. (*bouquets rouge*)

Fortunately, some stories from Métis people also illustrated the efforts taken by the Métis community in Victoria to connect in online spaces. In fact, some participants said the COVID-19 pandemic brought new opportunities to connect with Métis culture and community online:

Especially with COVID, We’ve really started connecting online with the Métis community here... even tonight the kids have a Zoom meeting, they were playing an online Scattergories game with the Greater Victoria Métis Nation and on the weekend they did a workshop where they made dream catchers…. (*lii tii’d mashkek*)

I’m super thankful for the community in Victoria. I know previous to the whole COVID outbreak they had a ton of like programs for culture and stuff… So they have a lot more programs available over technology, which is super nice. (*li grachaw*)

At the same time, participants also recognized that virtual platforms could not replicate in-person connection to community:

I think that that’s the biggest thing that I miss, especially having a hard time with life stuff and then not being able to gather is really tough. And you know, the virtual thing. Like we can do it but… you start to get sick of it after a while. And it’s definitely not the same. (*lii tii’d mashkek*)

Unfortunately, some participants noted that their experiences of isolation during and prior to the COVID-19 pandemic were shaped by intersecting experiences of poverty, disability, or chronic illness. For example, *lii groo zel* and *plaanten* reflected:

I’m probably less impacted by COVID-19 than most people because there’s nothing like having a chronic health condition… it’s isolating in and of itself. (*lii groo zel*)

My kid and I are pretty self-isolating people anyway so the extent of what we’ve had to deal with, it’s probably the same as it is for a lot of people in our situation. We’re living kind of below the poverty line. We kind of keep to ourselves anyway, so I don’t know that there’s anything specific to being Métis, to be quite honest with you. (*plaanten*)

Conversation with one participant who had previously contracted COVID-19 demonstrated how isolation from community and support networks can have serious consequences for Métis community members who are experiencing serious illness:

We had no one to get us groceries. Because of COVID, I didn’t meet that many people…There was no program to phone and say, ‘Hey, I need some groceries.’ Like, ‘Please help me.’ So that made me feel, very alone, very isolated, very not cared for living here. (*enn fleur daan loo*)

Although she has since recovered from COVID-19, the lack of care she received during her illness has left a lasting impact:

I feel more lonely, I guess. But only just this month, it’s really affecting me. But before that I was fine. I guess after my COVID experience... I’m still not over feeling so isolated. (*enn fleur daan loo*)

### COVID-19 and access to culturally safe services

Many participants identified specific challenges related to the availability of accessibility of culturally safe healthcare in Victoria, BC. Many described how these challenges intensified during the COVID-19 pandemic. Participants spoke about challenges they faced in accessing care that were common among the general population. For example, long wait times, lack of comfortable and/or safe waiting areas, inconsistent and confusing booking processes, and unreliable public transit were commonly mentioned throughout conversations. However, in addition to these common challenges, conversations with participants illustrated how the risk of contracting COVID-19 while accessing health services layered onto multiple pre-existing barriers, including discrimination from service providers and challenges securing reliable and safe transportation to get to medical appointments:

There’s a very real fear of getting COVID. A fear of discrimination. And then, of course, just the limitations of the healthcare system itself. And if you don’t have a vehicle, good luck. It’s very, very difficult to get from A to B. And when you have healthcare professionals that are under a lot of stress right now with COVID, the wait times are extended, and you can miss your connections for buses. You’re afraid to get into a cab. If you have someone going with you, they have to be within your bubble, and right now you’re not supposed to be extended outside of that bubble, so you’re relying on at least one point person, and the stress of that is a lot too. (*li pchi boom*)

Indeed, many participants spoke openly about their experiences with racism and discrimination within the healthcare system. Some participants, like *enn fleur di pwayzoon*, also described microaggressions that intersect sexism and racism: “I absolutely do get fetishized by men… for like looking mixed…. I have that ‘exotic look’ I’ve been told.” While experiences of racism and discrimination in healthcare are explored in-depth elsewhere (see Paul et al., 2023), it is also important to highlight the layers of sexism and racism. Adding further to these layers of oppression, another participant shared that she often is faced with people saying she is “lucky” to receive disability benefits; *enn fleur daan loo* shared her reactions to such comments: “They’d say, ‘Oh, well, you’re Métis. You’re a woman. And you have a disability. Oh my God, you’re so lucky.’ But really, they’re saying, ‘Screw you.’”.

For some Métis participants, sexism and agism shaped their negative healthcare experiences in more obvious ways than racism. For instance, *enn fleur di pwayzoon* spoke about being patronized and dismissed when accessing mental healthcare:

I don’t think it was my Métis heritage, but I think it was just being younger and when I became interested in my mental health because I wasn’t doing very well. I went to go get help and I was really talked down to and I don’t think that’s just a Métis experience, I think that’s actually common for women and I think it’s common for the younger women especially and women’s health is completely dismissed in a lot of ways. So, I think when I went to get help, it was like, ‘Yeah, just go on drugs,’ that was the response. And when I went to further investigate or ask more questions, it was just, like, I was just being annoying, you know or I wasn’t being open minded enough. It was just kind of like everything that I was being condescended towards, that I didn’t have the awareness or the intelligence or the knowledge in myself to know what I might need or be a part of an effective healthcare plan for myself. (*enn fleur di pwayzoon*)

For this participant, as well as many others, protective strategies in healthcare often include choosing not to disclose their identity as a Métis person. This was regarded as a privilege among Métis people who may pass for, or “look,” white. Another participant shared a similar experience of gender discrimination and agism, in mental healthcare:

I find because I think our concerns can be dismissed. Like mine was about having anxiety and stuff. I felt like if I was older I wouldn’t have been dismissed, you know? But I think because they were like, ‘Oh, you’re... you’re just young, like you don’t…’ like, ‘That’s just part of life. Like, don’t you…’ You know? So, yeah, I like to hear from people my age, how they’ve experienced the doctors as well. (*lii groo zel*)

Both *enn fleur di pwayzoon* and *lii groo zel* attribute these experiences to being young women, illustrating the ongoing issues with gender discrimination in healthcare settings. In addition to mental health, participants also highlighted particular issues with sexual health. Other participants shared that they were intentionally taught by their mothers that medical professionals may not believe them, and they were raised with self-advocacy skills to help them address potential experiences. For another participant, access to primary care has become limited due to a lack of providers who are culturally safe for Indigenous people, as well as competent and welcoming of transgender patients.

A common practical challenge discussed throughout many conversations was how precautions to limit transmission of COVID-19 have prevented Métis women, Two-Spirit, and gender-diverse people from bringing an advocate or support person to medical appointments. This was particularly concerning for participants who were seeking medical care due to a mental health crisis, major surgery, or experience of violence. For example, one participant described how COVID-19 protocols prevented her from bringing a support person for survivors of sexual assault to accompany her to the emergency room. As a result, she was left to navigate this process with unfamiliar health providers and without emotional support. Another participant described a similar experience, going on to describe how such protocols can impact Métis people experiencing a mental health crisis:

Right now because of COVID they’re not allowing anybody but the patient into like the emergency room. And so no matter who you are, you can’t have any support person. And just the way that everybody in a hospital deals with people, it’s almost conducive to the mental health problem getting worse. (*zayon faroosh*)

Overall, these stories illustrate that COVID-19 has intensified existing barriers to culturally safe services for Métis people in various ways. In several cases throughout participant interviews, these barriers prevented participants from accessing needed healthcare.

### Economic impacts of COVID-19

Responses touching on the economic impacts of the COVID-19 pandemic were varied and reflect the diversity within and across Métis communities. These stories also highlight the specific ways that Métis women, Two-Spirit, and gender-diverse people experience financial instability in distinct ways. Several participants said COVID-19 brought additional strain on their financial resources. In particular, Métis women who were studying at post-secondary institutions described financial strain due to the pandemic. For example, two participants shared:

It’s impacted my financial situation a lot. It was very difficult to find a job this summer. (*bouquets rouge*)

While it was nice, and I know that Canada’s commitment to the $1,250.00 for the student benefit was good, it wasn’t enough… Thankfully, with some extreme budgeting, I was able to make it work - but for a lot of people I know that wasn’t nearly enough. (*lii grenn bleu*)

Additionally, two participants described the added strain that illness had on their caregiver duties. One participant, *enn fleur daan loo*, said this about the expectations that were placed on her to care for her family while recovering from COVID-19, “He was getting mad at me for not doing the dishes. And then he realized, once he got sick, ‘Okay, this is what it’s like.’” Further participants like *li sayd* spoke generally about the added challenges from COVID-19 for single parents.

While several participants reported experiences of financial strain due to the pandemic, a small number of participants shared that the pandemic had inadvertently increased their household income. For instance, one participant shared that an unforeseen consequence of the COVID-19 pandemic has been an increase in online work opportunities and appreciation for creative work:

I think my creativity is being recognized more and I’ve been given more opportunities to rebuild. I don’t know how that happened. I don’t know if that’s just because we’re more online and the opportunities for online work have become more plentiful. (*enn fleur di pwayzoon*)

As well, two participants shared that government benefits distributed during the COVID-19 pandemic (such as the Canada Emergency Response Benefit and the BC Recovery Benefit) had provided a sense of financial stability with noticeable impacts on their quality of life. For example, *enn fleur di pwayzoon* was able to secure safe housing:

It also offered me the opportunity to finally move. I didn’t have a home for a long time. And because I couldn’t afford to move and because I was eligible for the CERB, I was able to actually move. So, yeah, so that was actually a good thing for me. (*enn fleur di pwayzoon*)

Similarly, *plaanten* shared, “It [COVID-19] gave me an income that I didn’t have…It’s like yeah, it’s been terrible and also wow, I have financial stability for the first time in three years” (*plaanten*). In this sense, both *plaanten* and *enn fleur di pwayzoon* felt that government benefits distributed during the COVID-19 pandemic had provided a sense of financial stability with noticeable impacts on their quality of life. These statements are striking and they highlight the systemic factors that create different economic conditions and outcomes for women and gender-diverse people, broadly. Gender-based discrimination and disproportionate responsibilities for unpaid care work are among the well-known factors that shape the economic experiences of women and gender-diverse people. As well, specific conditions that Métis women, Two-Spirit, and gender-diverse people experience—including disproportionate interactions with colonial systems (e.g. the child welfare system)—may amplify these challenges and increase financial instability.

## Discussion

Results from this study highlight the breadth of impacts that the pandemic has had on the lives of Métis women, Two-Spirit, and gender-diverse people in Victoria, including their ability to access needed health services and social services. The experiences shared by participants demonstrate that they continue to rely on Métis social kinship connections and cultural practices for their day-to-day wellness. These findings converge with Métis-specific research elsewhere, where Métis-led research with Métis women has illustrated the value of land-based practices and social connection in supporting Métis women’s well-being (Flaminio et al., 2020; Foulds et al., 2021).

Unfortunately, many strategies to mitigate the spread of COVID-19, such as physical distancing and stay-at-home measures, also disrupt cultural gathering practices which remain integral to lifeways for many Métis individuals and families. The unintended negative impacts of COVID-19 on the Métis community in Victoria are compounded by new and pre-existing gaps in the availability of and barriers to culturally safe health and social services. Barriers in accessing health and social services often result in Métis women, Two-Spirit, and gender-diverse people avoiding services, resulting in unmet service needs (Auger, 2019; Monchalin et al., 2020), which is deeply concerning in this time of global health crisis.

Our findings are echoed by emerging research pertaining to Métis people, access to healthcare, and COVID-19. For example, the stories and experiences shared by participants align with findings from the recent *In Plain Sight* report (Addressing Racism Review, 2021), which explores anti-Indigenous racism in BC healthcare. Results of this comprehensive review contribute further evidence to our finding that the COVID-19 pandemic has increased health service access barriers for Métis people in BC. Further, Métis people are experiencing disproportionate effects on their mental well-being during the pandemic compared to BC’s overall population (Addressing Racism Review, 2021). *In Plain Sight* also found that Métis women are disproportionately affected by poor health compared to their male counterparts, and although their health service needs are greater, they feel less safe than Métis men when receiving health services. Our findings indicate that Métis women, Two-Spirit, and gender-diverse people experience discrimination rooted in multiple, sometimes overlapping, oppressive structures which is evidenced through the unique and concerning experiences shared in their stories. 

Addressing the ongoing legacy of colonialism within healthcare systems is essential for providing culturally safe care and improving health outcomes for Métis women, Two-Spirit, and gender-diverse people during the pandemic and beyond. There are several examples of innovative, culturally safe, and community-based COVID-19 initiatives that are led by urban Métis women across Canada (Heck et al., 2021; Jones et al., 2020; Monchalin et al., 2019). The *Call Auntie Hotline* in Toronto, for example, is a culturally safe and community-led resource created by Métis/Cree midwife Cheryllee Bourgeois with support from a group of Indigenous birth workers and harm reduction advocates known as *the Aunties*. The *Call Auntie Hotline* provides COVID-19 information, resources, advocacy, referrals, self-assessment, self-isolation information, sexual and reproductive health facts, and any secondary support necessary as a result of COVID-19. Another example of innovative and culturally safe responses to COVID-19 is *The Breathe Project*, which was co-created at a grassroots level by two Métis artists, Lisa Shepherd and Nathalie Bertin (Johnson, 2020). *Breathe* began as an online gathering space to combat the isolation experienced by many Indigenous women during COVID-19, where community members were encouraged to create masks using materials from their diverse cultural traditions. *Breathe* grew into two traveling art exhibitions that toured Canada in 2023, helping to generate income for Indigenous artists while simultaneously nurturing community connections.

These initiatives demonstrate that Métis women, Two-Spirit, and gender-diverse people continue to carry knowledges and solutions that are vital to the well-being of their communities. Unfortunately, the perspectives and experiences of Métis people are often excluded from Indigenous-health research (Auger, 2019; Gmitroski et al., 2023; Wesche, 2013). For example, Canada’s federal and provincial governments have failed to carry out consistent health outcome monitoring for many Indigenous communities, other than First Nations people living on-reserve; consequently there is an absence of available data pertaining to COVID-19 rates among Métis populations (Heck et al., 2021). This lack of reliable, distinctions-based public health research and data relevant to COVID-19 has resulted in the exclusion of Métis women, Two-Spirit, and gender-diverse people in the design and implementation of national, provincial, and community-level COVID-19 responses (Jones et al., 2020). These compounding factors contribute to continued gaps in services, inequitable health outcomes, and systemic barriers for Métis communities in protecting the health and safety of their members.

## Conclusion

The stories shared within our conversations highlight the vital importance of culturally safe service options for Métis women, Two-Spirit, and gender-diverse people, especially in times of public health emergencies. To address inequitable access to health and social services among Métis populations, public health measures addressing COVID-19 must draw on the strengths that exist within our communities, as well as address the specific barriers to healthcare. Improving access to culturally safe health and social services by incorporating the experiences and expertise of Métis women, Two-Spirit, and gender-diverse people to support distinctions-based approaches to culturally safe service delivery is crucial to mitigating the disproportional negative impacts of the pandemic and improving overall health outcomes within Métis communities across Canada.

## Contributions to knowledge

What does this study add to existing knowledge?

• This study highlights some of the impacts that the pandemic has had on the lives of Métis women, Two-Spirit, and gender-diverse people in Victoria, BC.
• Study results demonstrate how the COVID-19 pandemic has imposed new and exacerbated challenges in accessing culturally safe health and social services for Métis women, Two-Spirit, and gender-diverse people.

What are the key implications for public health interventions, practice, or policy?

• To address inequitable access to health and social services, Métis people must have meaningful input into pandemic response measures and health service delivery in their local communities.
• There is a need for further Métis-led research and collaboration to support evidence-based pandemic responses and locally tailored approaches to culturally safe service delivery.

### Supplementary information

Below is the link to the electronic supplementary material.

Supplementary file1 (DOCX 21 KB)

## Data Availability

The data that support the findings of this study are available on reasonable written request from the corresponding author. The data are not publicly available due to their containing information that could compromise research participant confidentiality and consent.
