# Supplementary material for: “I’m still not over feeling so isolated”: Métis women, Two-Spirit, and gender-diverse people’s experiences of the COVID-19 pandemic
Source: Can J Public Health. 2024 Jan 17;115(2):199–208. doi: 10.17269/s41997-023-00849-3 (PMC11006636; doi:10.17269/s41997-023-00849-3)
Supplement: Supplementary file 1 — Supplementary file1 (DOCX 21 KB) [file 41997_2023_849_MOESM1_ESM.docx]

**Interview Guide**

*(After you have reviewed the consent form)*

*Thank you for meeting with me today! Today I would like to have a conversation about your perspectives on Métis identity and your experiences with health services in Victoria. The goal of this research study is to address the health service gap for the Métis community in Victoria. Remember that you can take breaks at any time, and that you can skip any question that you don’t want to answer. If you have any questions, you can stop me at any time. I estimate that today’s interview will take about 1 hour. Do you have any more questions about the consent form? Do you have any questions in general? Are you ready to get started?*

1. **How would you describe yourself?**

Based on how the question is answered above, a supplementary question is…

- 1. What makes up your identity?

1. **What does being Métis mean to you?**

Based on how the question is answered above, a supplementary question is…

- 1. When was the first time you knew you were Métis?

1. **Can you describe your experiences with health services in Victoria?**

Based on how the question is answered above, supplementary questions are…

1. Do you have any experiences with ***Indigenous specific*** health services in Victoria?
2. What are your experiences with ***non-Indigenous specific*** health services in Victoria?
3. **Can you describe a time that you felt you did not receive health services you needed? What happened? What was your unmet need?**

*This next question will be surrounding your experiences with racism and/or discrimination. We often hear about people being treated poorly or unfairly because they are Métis. Remember that you can take breaks at any time, and that you can skip any question that you don’t want to answer. If you have any questions you can stop me at any time.*

1. **Can you think of a time that you have been treated poorly or unfairly because you are Métis? How did this impact your overall health and wellbeing?**

*Next, we wish to ask one question related to COVID-19. Given that the COVID-19 pandemic has impacted the larger Métis community in unique ways, we were wondering about your experiences during the pandemic.*

1. **Can you describe how COVID-19 has impacted you?**

*I want you to* *imagine a relationship with a health and/or social service provider in which you feel comfortable, respected and able to be yourself.*

1. **How would it look?**
2. **What are the things that the service provider does to make you feel comfortable and respected and able to be yourself?**
3. **What about the space where the care is being provided? How does it look or feel?**

Based on how the question is answered above, a supplementary question is…

- 1. What are the things in the space that make you feel comfortable and respected and able to be yourself?

*The last question surrounds how you would like to see the results from this conversation used. This is an opportunity for a co-developed idea between the research team and yourself to develop something that is relevant to the Métis community in Victoria.*

1. **How would you like to see the findings from this conversation used?**

*Thank you for participating in this interview. Now that your interview is complete, please confirm the following questions below* (Interviewer will make necessary updates on the consent form during this time if required with the participants approval).

1. *Do you want to be sent a copy of your transcript for review and approval? Yes / No;*
   1. *If yes, what is the mailing address you would like your transcript mailed to? _____________________________________________________________;*
   2. *If no, are you comfortable with us using your transcript as is in its current state? Yes / No;*

*2) Do you consent to having direct quotations from your transcript used for publication? Yes / No;*

*Now that the interview is complete, our team will review your transcript for themes. Once this is complete, your transcripts will be mailed to you within two weeks from the date of this interview upon your request for verification and corrections with a note of the themes the research team found. If you wish to have your transcript mailed to you, I will follow up with you by phone to discuss verification and/or corrections in your transcript.*

*If you require further information or have problems concerning the research study, please contact the principal investigator at* [*rmonchalin@uvic.ca*](mailto:rmonchalin@uvic.ca) *or* [*250 472 4431*](tel:250%20472%204431) *during work hours.*
